# Perforated IUD as Atypical Cause of Recurrent Urinary Tract Infection: A Case Report

**DOI:** 10.1155/criu/8941217

**Published:** 2025-10-22

**Authors:** Sophia Ford, Samantha Levine, Aaron Baer, Kristie Lou, Eric Ballon-Landa

**Affiliations:** ^1^Division of Urology, Department of Surgery, Saint Louis University School of Medicine, Saint Louis, Missouri, USA; ^2^Division of Minimally Invasive Gynecologic Surgery, Department of Obstetrics and Gynecology, Saint Louis University School of Medicine, Saint Louis, Missouri, USA

**Keywords:** case report, Lippes Loop intrauterine device, recurrent urinary tract infection, retained foreign body, robotic surgery

## Abstract

Clinicians faced with common diagnoses such as pelvic pain or recurrent urinary tract infection (rUTI) may not consider less frequent etiologies, such as a delayed erosion of an intrauterine device (IUD). Given that modern IUDs are not approved for more than 10 years, early or midcareer clinicians unfamiliar with the earlier versions of these devices may not consider that a long-retained foreign object may be the source of symptoms in an older woman. We report a case of an IUD placed 50 years ago that eroded into the bladder, causing recurrent UTI and pelvic pain. Thankfully, minimally invasive laparoscopic techniques available now—to date unreported—enable surgical management while limiting morbidity, which is especially important in an elderly population.

## 1. Introduction

Intrauterine devices are a highly utilized and effective form of pregnancy prevention, with failure rates as low as 0.02% [1, 2]. In the United States, two forms of IUDs are FDA approved for long-term contraception: the copper-containing IUD and levonorgestrel-containing IUD [2], both of which are “T”-shaped devices that insert into the endometrial cavity. Before this “T”-shaped design became popular, earlier models utilized different designs, some of which are still used internationally [3]. The Lippes Loop IUD, first released in 1962, was the first model to utilize the novel double-S design. This flexible, snake-like shape was thought to fit the contours of the uterine cavity in a way that would reduce the risk of expulsion [4]. This was a nonhormonal option marketed for long-term use. Therefore, it is not uncommon for recipients of the Lippes Loop IUD to retain their device for decades, whether purposefully or because their device had been forgotten [5, 6].

Due to their longevity and popularity, adverse events associated with IUDs have been reported in the literature. One such complication is the migration of the device into nearby anatomical structures, which is most likely to occur in the first year after placement [7, 8]. The most common locations of IUD migration include the intestines, bladder, and omentum. Risk factors include prior uterine surgery (e.g., cesarean section), hypoestrogenic states (breast feeding and menopause), or proinflammatory states that can weaken the uterine walls [8]. Migration with associated expulsion occurs in as many as one to two cases per 1000 insertions [7]. Among those whose IUD perforates surrounding structures, most will complain of pelvic pain; however, up to one-third can be asymptomatic.

Whether early or delayed, IUD migration has several clinical implications that should be considered by clinicians. The first is loss of contraceptive efficacy, which would be a risk for patients with complete erosion outside the uterus [9]. The second is the implications for the structures involved in the erosion. In the urinary tract, this can lead to pelvic pain, urinary tract infection, gross hematuria, or urinary stone disease. In the bowel, the implications may include GI distress, constipation, diarrhea, and rectal bleeding.

While there are many etiologies of IUD migration into surrounding structures, it remains an uncommon event. Nevertheless, various risks are associated with inserting a foreign body into the uterus. In cases involving multiple organ systems, laparotomy has been reported as the preferred surgical intervention; however, the increasing dissemination of robotic-assisted laparoscopic surgery has facilitated the safe completion of more complex pelvic surgery while maintaining a minimally invasive approach.

We present a novel case of minimally invasive (robotic-assisted laparoscopic) surgical removal of a retained Lippes Loop IUD, hysterectomy and bilateral salpingo-oophorectomy, and cystorrhaphy in an 81-year-old patient with myometrial and bladder perforation.

CARE guidelines were utilized in the development of the manuscript. Written informed consent for publication was obtained from the patient, including the utilization of deidentified images.

## 2. Patient Information and Assessment

An 81-year-old female with a history of hypertension, Type 2 diabetes mellitus, fatty liver disease, and rUTI presented to our urology clinic for surgical consultation regarding the removal of a retained IUD that had been placed 50 years prior. The patient had a Lippes Loop IUD placed in 1974 and since that time had developed a known myometrial and bladder perforation (Table 1).

The patient started developing rUTI in December 2020, with several documented cases of *Staphylococcus aureus*, *Klebsiella pneumoniae*, and *Proteus mirabilis* in the urine. These episodes were associated with urinary frequency, urgency, dysuria, and sometimes hematuria. She also reported a feeling of vaginal pressure that was present only intermittently and worsened with physical activity. She denied vaginal bleeding since menopause in her 40s, denied urine leakage per vagina, and denied any history of pain with penetration. She presented to urogynecology for rUTI and pelvic pain. Uterine perforation of this patient's IUD, without bladder perforation, was retrospectively noted on computed tomography in 2016, 4 years prior to the onset of her symptoms. Her last normal imaging without any perforation was 2010.

**Figure 1:**
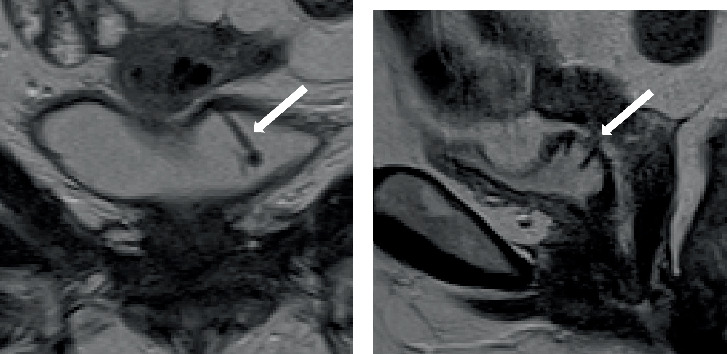
Preoperative MRI imaging demonstrating Lippes Loop IUD curls protruding into the bladder lumen in (a) coronal and (b) sagittal views (white arrows).

During her subsequent evaluation, the IUD was noted to perforate the bladder wall during clinic cystoscopy in May of 2024. A subsequent MRI revealed that the IUD had perforated the bladder lumen in at least three different areas, extending through the myometrium of the mid uterine body through the cervix anteriorly (Figure 1). Given her symptoms and these findings, the patient was recommended to undergo removal of the foreign body using a robotic-assisted laparoscopic approach to partial cystectomy and concurrent hysterectomy versus hysterorrhaphy. Alternatives such as observation were not desired given her symptoms, and an endoscopic procedure was not deemed feasible given the extent of her perforation and multiorgan involvement.

## 3. Intervention and Outcomes

**Figure 2:**
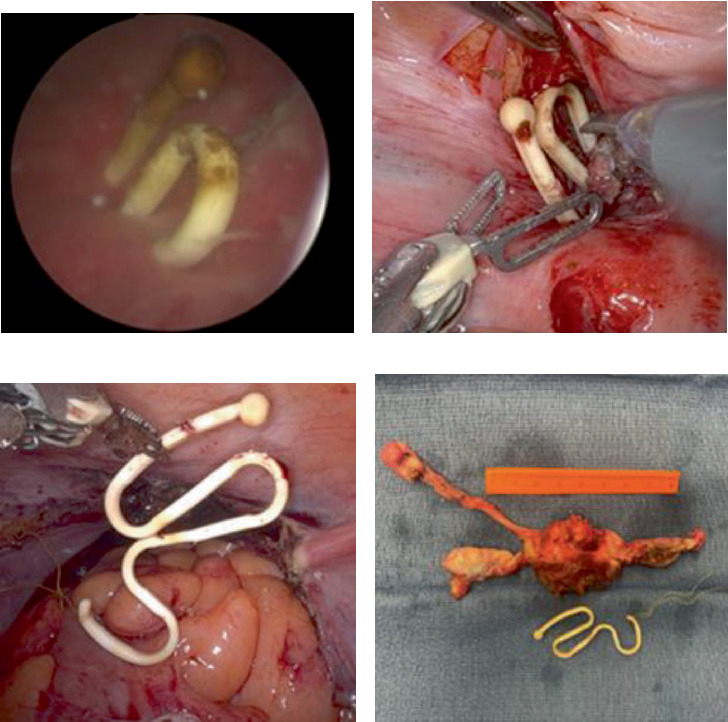
Intraoperative images demonstrating views of retained Lippes Loop: (a) cystoscopic image prior to removal, (b) robotic image after intravesical component removed, (c) robotic image after complete bladder and uterine extraction, and (d) surgical specimen and foreign body.

The procedure began with cystoscopy. The foreign body was visualized (Figure 2a), and the ureteral orifice, just inferior to the foreign body, was cannulated with a 5 French open-ended catheter to facilitate ureteral identification. Upon laparoscopic abdominal entry, a narrow band of scar tissue was noted between the uterus posteriorly and the bladder trigone anteriorly. The plane between these two structures was dissected, and the foreign body was visualized (Figure 2b). A proximal cystotomy was created and used to dissect the bladder wall circumferentially until the foreign body was completely excised from the bladder mucosa. The left ureteral orifice was close but uninvolved with the resection.

The gynecology team subsequently completed a supracervical hysterectomy. The uterus was inspected, and the foreign body was found to perforate the uterus at multiple points; the IUD was then removed from the uterus with gentle traction (Figure 2c). It was noted to be fully intact with the strings attached, and it was removed from the abdomen. The surgical team did not feel that hysterorrhaphy would be appropriate given that multiple points of uterine perforation were noted and were extensive. The uterine tissue integrity was poor because of her age, and the risk of uterine fistula to surrounding structures was felt to be high. A supracervical hysterectomy with bilateral salpingo-oophorectomy was completed, and the uterus was removed from the abdomen (Figure 2d).

The cystotomy was then reapproximated and closed in two layers with absorbable suture in running fashion. Both ureteral orifices were visualized through the cystotomy with clear urine effluxing. A water leak test was performed with no leak appreciated.

Postoperatively, the patient did well without any complications. She was discharged on the day after surgery. She achieved pain control, ambulated, and tolerated a regular diet prior to discharge. A catheter was left in place for 1 week, and the postoperative cystogram prior to removal demonstrated no contrast extravasation. Pathologic analysis of the hysterectomy specimen demonstrated benign inflammatory changes.

## 4. Discussion

This case highlights an unusual cause of rUTI and the successful removal of a migrated IUD from the bladder using a minimally invasive technique. Perforation of the device is a known risk of IUD placement. The first typical sign of migration is the inability to visualize the IUD threads from the cervix on physical exam, which would then prompt the clinician to recommend a transvaginal ultrasound to visualize the device. Despite her extensive perforation, our patient had visible IUD threads via the cervix throughout her entire treatment course from insertion to surgical removal. As such, clinicians caring for patients with long-term foreign body retention should retain a clinical suspicion for migration and consider pelvic imaging in the setting of complaints such as pelvic pain, uterine bleeding, hematuria, or rUTI.

The minimally invasive robotic approach offered several advantages over other techniques in this case, which offered additional complexity in that two organ systems (the bladder and the uterus) were equally involved. First, a minimally invasive approach rather than open surgery allowed for rapid recovery and return to regular function; the patient was admitted overnight for observation but required very little inpatient care. The robotic-assisted approach over traditional laparoscopy allowed the surgical team to address the complexity of her erosion successfully without requiring ureteral reimplantation, given the close proximity of her erosion to the left ureteral orifice and ureter. Complex suturing and bladder reconstruction were relatively straightforward with the addition of the robotic platform.

A case-based approach is prudent in these situations, as clinicians have had success with endoscopic, laparoscopic, and open techniques as published in the literature [7–9]. Prior studies have demonstrated experiences where only one organ system may be involved, as with complete erosion through the uterus into surrounding structures [9–11]. In these cases, the uterus may not require any treatment, and pure abdominal laparoscopy, endoscopy, or cystoscopy with laser may offer sufficient means to complete surgery successfully [7, 9–11].

Given that our patient's strings remained in place, migration of the device was investigated in this patient due to her rUTI with associated lower urinary tract symptoms. Limited imaging studies confirmed an appropriately positioned IUD in 2010, projection of the IUD through the uterus in 2016, and finally a perforated bladder in 2024. This timeline provides a conceivable consistency with the inception of the patient's urinary symptoms in 2020.

These findings are limited by several factors. The results lack generalizability due to the case report format. Nevertheless, this case offers guidance to urologists and gynecologists faced with similar clinical scenarios. Future studies with larger patient populations would help to define characteristics better. Although early IUD migration occurs more frequently, clinicians will see fewer of these delayed complications as the older devices are phased out among younger generations of women.

Since the reintroduction of contraceptive IUDs to the market in 1988 (after a brief hiatus due to safety concerns), recommendations for duration of implantation have been generally limited to a maximum of 10 years. Subsequently, late expulsion events such as the one in this case are expected to become even more infrequent. This case serves as an excellent reminder of why treatment recommendations are constantly under review and why close adherence to current guidelines helps protect patients from adverse events. Clinicians treating patients with a history of foreign body insertion—especially if remote—should expedite imaging workup in order to rule out device-related causes of urinary tract symptoms.

## Figures and Tables

**Table 1:** Clinical timeline of IUD placement, imaging, and symptoms.

**Date**	**Event**
1974	Lippes Loop IUD insertion
2010	Imaging without perforation
2016	Asymptomatic uterine IUD perforation
12/2020	Development of recurrent UTI and pelvic pain
5/2024	Cystoscopy: IUD perforation into the bladder
8/2024	Robotic-assisted IUD removal, cystorrhaphy, and supracervical hysterectomy

## Data Availability

Data sharing is not applicable to this article as no datasets were generated or analyzed during the current study.

## Patient Perspective

The surgery was successful with fewer complications than were possibly anticipated. I was out of bed and using the bathroom on the day of surgery. While the Foley catheter was cumbersome, it was necessary because of bladder involvement to tolerate what I needed to. My course of recovery was tolerable, and I was ambulating very quickly. I remained home for 3 weeks and was able to do personal hygiene from Day 2. I am extremely grateful to my care team. It has been 8 weeks since my surgery and my quality of life has not been compromised. I have resumed bowling with a 12-pound bowling ball, driving, and all activities that I did before surgery. It was a great relief that I no longer had this foreign object in my body. I hope that this perspective will help someone in the future who may develop a similar problem.
